# Dr. Challen

**Published:** 1833

**Authors:** 


Joseph Challen, M. D. of *Lexington, Ky.* This gentleman was a graduate of the Transylvania School, and a native of Lexington; but commenced his professional career in this city, whence he returned to Lexington a few months since, leaving behind him many friends both in and out of the profession. He fell a victim to the Cholera, during its late visitation at Lexington, June 11th, in his 30th year.

